# Quantifying causal effects from observed data using quasi-intervention

**DOI:** 10.1186/s12911-022-02086-z

**Published:** 2022-12-21

**Authors:** Jinghua Yang, Yaping Wan, Qianxi Ni, Jianhong Zuo, Jin Wang, Xiapeng Zhang, Lifang Zhou

**Affiliations:** 1 School of Computer Science, University of South China, Hengyang, China; 2 Hunan Provincial Base for Scientific and Technological Innovation Cooperation, Hengyang, China; 3 Hunan Cancer Hospital, Changsha, China; 4 School of Nuclear Science and Technology, University of South China, Hengyang, China; 5 The Third Affiliated Hospital of South China University, Hengyang, China

**Keywords:** Causal effect, Intervention, Do-algorithm, Quasi-experimental design, Quasi-intervention

## Abstract

**Background:**

Causal inference is a crucial element within medical decision-making. There have been many methods for investigating potential causal relationships between disease and treatment options developed in recent years, which can be categorized into two main types: observational studies and experimental studies. However, due to the nature of experimental studies, financial resources, human resources, and patients' ethical considerations, researchers cannot fully control the exposure of the research participants. Furthermore, most existing observational research designs are limited to determining causal relationships and cannot handle observational data, let alone determine the dosages needed for medical research.

**Results:**

This paper presents a new experimental strategy called quasi-intervention for quantifying the causal effect between disease and treatment options in observed data by using a causal inference method, which converts the potential effect of different treatment options on disease into computing differences in the conditional probability. We evaluated the accuracy of the quasi-intervention by quantifying the impact of adjusting Chinese patients’ neutrophil-to-lymphocyte ratio (NLR) on their overall survival (OS) (169 lung cancer patients and 79 controls).The results agree with the literature in this study, consisting of nine papers on cohort studies on the NLR and the prognosis of lung cancer patients, proving that our method is correct.

**Conclusion:**

Taken together, the results imply that quasi-intervention is a promising method for quantifying the causal effect between disease and treatment options without clinical trials, and it could improve confidence about treatment options' efficacy and safety.

## Background

In biomedicine, causal inference often relies on the framework of counterfactual reasoning. For example, given an observed target image with lesions and a reference image without lesions in the corresponding region, what would the features of the target image look like if the lesions were removed? Through such comparison or thinking, researchers can quickly estimate the causal relationship, find the answer to the question, and relieve the suffering of the patient. Counterfactuals are located at the top of the ladder of causation [1], which is Judea Pearl's ladder of three different levels based on cognitive ability, with the remaining two levels being association and intervention. Under counterfactual theory, everyone has a potential outcome in different states, and by comparing the outcomes of individuals in different states, the causal effect of treatment on the outcome can be obtained [2]. However, in practice, counterfactuals are never observed because a single person (or group) cannot choose different states at the same time and place, so how to use observational and experimental data to extract information about counterfactual scenarios becomes the focus of scholarly research.

The most common experimental strategy is to conduct a randomized controlled trial (RCT). Because of the randomized nature of RCTs, the subject and their "counterfactual" counterpart have the same or similar values for the confounding variables, except for the relevant condition variables, to approximate the potential outcome [2]. However, RCTs are expensive, time-consuming, and ethically concerning, making many experiments a luxury [3]. In statistics, researchers are fond of viewing the counterfactual causal inference problem as a missing data problem, i.e., solving for the potential outcomes corresponding to different individuals in different states. Common methods for inferring missing data include matching [4] and linear regression. Matching methods refer to finding several pairs of individuals who match well on all other variables except the target variable, and then we can calculate the missing data based on this matching relationship. However, there will always be special cases in the data that cannot be matched. Linear regression methods assume that the data come from a random source at some location, then use standard statistical methods to find the best-fitting straight line for the data, and finally use padding techniques to resolve the missing data. Although this method cleverly calculates an approximation of the missing outcome, the number is not a potential outcome and cannot be used to make counterfactual causal inferences. The reasons for this are as follows: on the one hand, the method is data-driven rather than model-driven by nature; on the other hand, and more importantly, there is simply no situation where a Tier 1 method of the ladder of causation can solve the counterfactual problem (Tier 3). In the ladder of causation, the three levels correspond to complex causal problems, and each level holds power beyond the reach of the next. Thus, the data cannot tell us that we are in a counterfactual or fictional world or what will happen.

In this paper, we present a new experimental strategy called quasi-intervention for quantifying the causal effect between disease and treatment options in observed data by using a causal inference method, which converts the potential effect of different treatment options on disease into computing differences in the conditional probability. With the given observed data, quasi-intervention takes advantage of a quasi-experimental design (QED) [5–7] to determine the causal relationship between variables and uses a sign test to ensure the reliability of the results. To quantify the causal effect between disease and treatment options, with the help of hypothetical interventions [8–11], we implemented different treatment options for the patients and compared the difference between the means. We evaluated the accuracy of the quasi-intervention by quantifying the causal effect between the NLR and OS (169 lung cancer patients and 79 controls) among Chinese patients. Our results showed that quasi-intervention could compute OS well corresponding to the average causal effect (34.4%) under variable NLR intervention conditions. The result agrees with the literature findings [12–20], which consist of nine papers on cohort studies on the NLR and the prognosis of lung cancer patients, proving that our method is correct.

## Methods

### Determination of causal relationships between variables

Correct causality is the primary premise of this study and the guarantee of the correct conclusion. If intervening variable (*X*) and outcome variable (*Y*) have a purely correlational relationship, rather than causality, then this will lead to poor business decisions. In this method, we used a QED to infer causality from observed data. We supposed we had a pre-processed (e.g., factorization) dataset *Q*, which consists of epidemiological information, such as age, sex, and clinical records, for all patients who do not intersect. To make better counterfactual causal inferences, the following assumptions were made about the study population: there is no crossover treatment effect between individuals; all individuals are treated to the same extent; the assignment of treatment is independent of the potential outcome; and the probability of assignment of treatment is nondeterministic for all individuals. We will consider here in detail that the sample size is larger than 20. The specific experimental steps are as follows:

- We defined a matched set of pairs *P* as follows. Let *T* (*T* ⊆ *Q*) be the set of all patients who have been treated. Then, we picked the intervention patient *u*(*u ∈ T*) and paired them with a patient *v* picked uniformly and randomly from a noninterventional patient set *D*, which means *u* and *v* have similar age, same sex, similar clinical notes and so on.
- For each pair *(u, v) ∈ P*, we assigned *outcome (u, v)* to + 1 if *Y*_*u*_ (patient *u* corresponding to *Y*) was larger than *Y*_*v*_, -1 if the outcome variable of *Y*_*u*_ was smaller than *Y*_*v*_, and 0 otherwise.
- The matching algorithm’s net outcome (δ) can be viewed as the difference between *Y*_*u*_ and *Y*_*v*_. The positive value of δ provides strong evidence of the causality between X and Y, while a negative value provides negative evidence.
  1 $$value(\delta )=\frac{\sum_{\left(u,v\right)\in P}outcome\left(u,v\right)}{\left|P\right|}*100\%$$

The QED obtained a causal effect between *X* and *Y* by controlling for observable confounders in the data. For the accuracy and completeness of the trial, the subsequent hypothetical intervention continued to explore the causal effects of the two variables under unknown confounding conditions. The fusion of the two methods overcomes their respective limitations and enhances the credibility of the experimental results.

To confirm the reliability of the result, we also used the sign test to determine whether our result was statistically significant. We formulated *H*_*0*_, which states that *X* has a null significant impact on *Y*, and let *H*_*1*_ be the alternative hypothesis in which it was assumed that *X* has an impact on *Y*. The number of positive or zero values of *outcome (u, v)* corresponded to *m* and *n*, respectively. After the removal of matching pairs with the same treatment effect, the sample size(*s*) was |P| − n. The measurement data *m* obeys an approximate normal distribution with a mean (μ) of $\frac{1}{2}s$ and a variance (σ) of $\frac{\sqrt{s}}{2}$. The significance level was set at an α of 0.05, and therefore, the statistic(*Ζ*) is $\frac{m-\mu }{\sigma }$. The null hypothesis H_0_ is rejected when *Ζ* > *Z* $\alpha /2$.

### Evaluating the effect of interventions

The chi-square test was used to compare the relationship between the clinicopathological data between groups with a given state *x* of *X* as a cut-off value. Kaplan–Meier univariate survival analysis and the log-rank test were used to analyse the survival of different patients, and the factors with statistical significance (P < 0.05) in the univariate analysis were independent factors affecting the prognosis of patients.

**Fig. 1:**
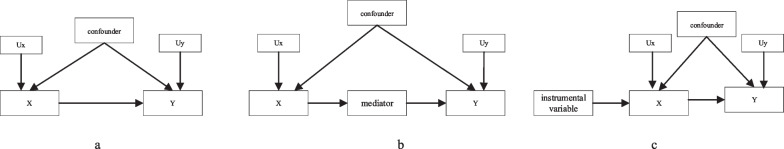
Three types of causal graphs. A confounding factor in the data (**a**), *X* can exert an indirect effect on *Y* through an intermediary variable (**b**); an instrumental variable is found for replacement studies (**c**). U_x_ and U_y_ are exogenous variables, representing any location or random effect that can affect the relationship between endogenous variables

There are three basic types of junctions in the causal graph: chain, fork, and collider. Through analysis, we can see that *X* and *Y* can only have the following three forms of causal diagrams (among them, “fork” can be divided into two types, and “collider” cannot form a bivariate causal diagram). These disturbance terms (e.g., U_x_, U_y)_ in Fig. 1, which are mutually independent, arbitrarily distributed random disturbances, represent exogenous factors that the investigator chose not to include in the analysis.

- A potential confounding factor is identified from the observed data, which means that the confounder blocks all backdoor paths from *X* to *Y* and is not a descendant of *X*. This is illustrated in Fig. 1a.
- If there are no obvious confounding factors in the observed data but a mediator (*W*) can be found to transmit the effect of *X* on *Y*, which means that all causal paths from *X* to *Y* pass through *W*, there is no unobstructed backdoor path from *X* to *W*, and all backdoor paths from *W* to *Y* are blocked by *X*. This is illustrated in Fig. 1b.
- If we are willing to accept the assumption of linearity or monotonicity, then an instrumental variable can be used to estimate the intervention effect (assuming the variable can be present in the data). Instrumental variables are required to affect *X* and not (directly) affect *Y*, as illustrated in Fig. 1c.

Supposing that we have the structure of a causal graph *G*, where some nodes are observable and others are not. Our main goal is to progressively reduce the expression $P(y|do(x))$ to an equivalent expression containing the standard probabilities of the observations. Notably, $P(y|do(x))$ stands for the probability of achieving a yield level of $Y = y$ given that the treatment is set to level $X = x$ by external intervention. It can be further stated that evaluating the effect of intervention involves computing the average causal effect (ACE):

$$P(Y |do(X = x^{\prime})) - P(Y |do(X = x))$$

where *do(.)* set *X* to a value, e.g., (*x* + 1). This intervention is equivalent to removing *X* from the influence of the old functional mechanism $X=f({pa}_{x},\varepsilon )$ and placing it under the influence of a new mechanism that sets its value to x + 1 while keeping all other mechanisms undisturbed. Clearly, an intervention $do(x)$ can affect only the descendants of *X* in *G*. The *do* operation allows the intervention effect to be obtained without the actual intervention, the counterfactual answer to be obtained, and thus the causal effect to be ascertained. The intervention not only replaced the causal mechanism linking X to its preintervention parents with a new mechanism *X* = *x* but also gave us a new manipulated graph. Interventional distributions (such as P (*Y* |*do*(*X* = *x*)) are conceptually quite different from the observational distributions (such as $P (Y |X=x)$). Because the latter does not have the *do*-operator, we can observe data from the dataset without carrying out any experiment. With the aid of the manipulated graph and the *do-algorithm* [8, 10, 11, 21], we eliminate the *do* operation in $P(Y |do(X = x))$, which represents hypothetical intervention and cannot be obtained from the dataset.

A causal relationship model characterized by graph *G* is identifiable, which has been demonstrated in Ref. [3]. This means that in a finite sequence of transformations, the causal relationship *Q* can be reduced to a check-free, probabilistic expression involving the observed quantity according to the *do-algorithm*. The derivation process is as follows: the probability distribution is first expanded according to a Bayesian formula, and then the expression is appropriately added, deleted, or replaced according to the *do-algorithm*, and the process is iterated until the expression no longer contains the *do* operation. It is noted that the experiment assumes that interventions are local and the global Markov assumption is true in the causal graph.

The *do algorithm* is described as follows:

*G* is the direct acyclic graph, *X*, *Y*, *Z*, *W* are any sets of variables in *G*, and *P* is the probability distribution. We use G $\overline{x}$(G $\underline{x}$, respectively) to denote that all arrows pointing to (emerging from, respectively) node *X* are deleted in *G*, and *Z(W)* is the set of *Z* nodes that are not ancestors of any *W* node in G $\overline{x}.$

1. Insertion/deletion of observations
  $$P\left(y|do\left(x\right),z,w\right)=P\left(y|do\left(x\right),w\right) \mathrm{if }(\mathrm{Y }||\mathrm{ Z}|\mathrm{ X},\mathrm{W}){{\rm{G}}_{\overline x }}$$
2. Action/observation exchange
  $$P\left(y|do\left(x\right),do(z),w\right)=P\left(y|do\left(x\right),z,w\right) \mathrm{if }(\mathrm{Y }||\mathrm{ Z}|\mathrm{ X},\mathrm{W}){{\rm{G}}_{\overline x \underline z}}$$
3. Insertion/deletion of actions
  $$P\left(y|do\left(x\right),do(z),w\right)=P\left(y|do\left(x\right),w\right) \mathrm{if }(\mathrm{Y }||\mathrm{ Z}|\mathrm{ X},\mathrm{W}){{\rm{G}}_{\overline x \overline {z\left(w\right)} }}$$

**Fig. 2:**
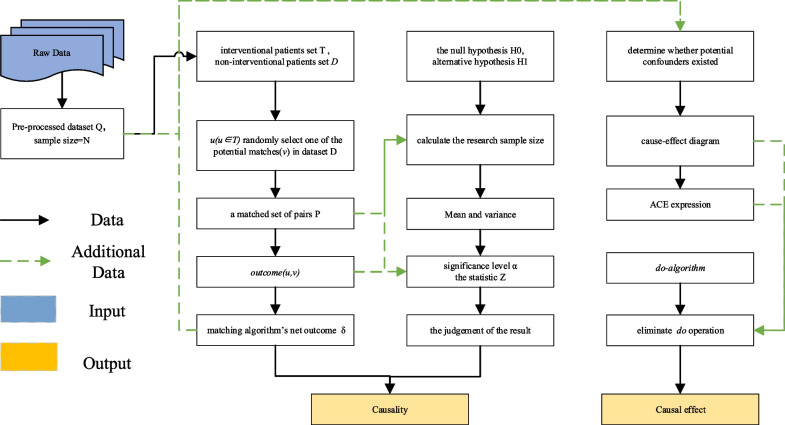
Experimental Flowchart of the Quasi-intervention

If we cannot find a way to estimate $P (Y | do(X))$ from the data in rules 1 to 3, then the solution does not exist for this problem. In this case, we realize that we have no choice but to run a RCT. In addition, it tells us what additional hypotheses or experiments could make the causal effect change from nonestimable to estimable for a particular problem. According to the derived causal diagram and *do-calculus*, we can eliminate the *do* operation in the ACE and quantify the effect of interventions between X and Y. The experimental workflow is shown in Fig. 2.

## Results

### Context of the study

Lung cancer is the most common form of cancer, with the highest morbidity and mortality in most countries [22–28]. The neutrophil-to-lymphocyte ratio (NLR) has been confirmed as an essential indicator of cancer prognosis and a risk of cancer metastasis in patients with lung cancer, and a high NLR was associated with poor overall survival (OS) [29–32]. However, most current studies reveal only a correlational relationship between the NLR and OS rather than a causal effect. Our study aimed to identify a causal relationship of the NLR with OS by quasi-intervention and quantify the impact of the NLR on OS, which contributes significantly to elucidating the cause of cancer and clinical treatment.

### Data source

Lung cancer patients who were treated in the Affiliated Nanhua Hospital, University of South China, and the First Affiliated Hospital of the University of South China from January 2012 to December 2017 were selected from the experimental dataset as the research participants. A summary of 169 Chinese lung cancer patients’ demographics is shown in Tables 1 and 2. Table 3 shows the peripheral leukocyte levels in lung cancer patients and normal subjects. All patients had no other history of malignant disease, and samples were collected before treatment, such as chemoradiotherapy, radiotherapy, and other treatment samples. One week before surgery, we identified the individual’s Karnofsky Performance Status (KPS) score. In addition, the anticoagulant tube was used to take 2–3 mL of fasting peripheral venous blood from each eligible patient, which was stored at 4 °C and examined within 1 h. In addition to patient demographics (including age, sex, date of diagnosis, smoking status, clinical stages, neutrophil count, KPS score, and lymphocyte count), the data collected included 79 healthy controls with normal lung condition from the physical examination centre in the Affiliated Nanhua Hospital, University of South China. All patients were followed until December 2018 by regular outpatient reviews and telephone. We extracted anonymized patient records from the electronic patient files. All patients who participated in the present study signed informed consent before the experiment, which was approved by the South China Ethics Committee. The experiment assumed that the neutrophil and lymphocyte count in the patient were according to the patient’s condition; the researchers did not deliberately interfere with the change.

**Table 1:** Comparison of clinicopathological characteristics of lung cancer patients in the high NLR group and low NLR group

Characteristic	All patients (n = 169)	Higher NLR	Lower NLR	X2	P value
Age, n (%)				0.001	0.974
≤55	43 (25.44)	10	33		
>55	126 (74.56)	29	97		
Sex, n (%)				2.174	0.14
Male	130 (76.92)	35	95		
Female	39 (23.08)	6	33		
KPS score, n (%)				8.131	0.043
60	11 (6.5)	3	8		
70	45 (26.63)	15	30		
80	93 (55.01)	43	50		
90	20 (11.83)	3	17		
Histology, n (%)				1.189	0.756
Adenocarcinoma	73 (43.2)	19	54		
Squamous cell carcinoma	77 (45.56)	16	61		
Small cell carcinoma	17 (10.06)	4	13		
Other	2 (1.18)	0	2		
Disease stage				2.276	0.517
I–II	12 (7.1)	4	8		
III–IV	157 (92.9)	35	122

**Table 2:** Univariate analysis of patient survival

Characteristic	All patients (n = 169)	Median OS	P value
Age, n (%)			0.204
≤55	43 (25.44)	28.9	
>55	126 (74.56)	26.29	
Sex, n (%)			0.713
Male	130 (76.92)	26.77	
Female	39 (23.08)	27.54	
Smoking status, n (%)			< 0.001
Never	124 (73.37)	26.86	
Ever	45 (26.63)	19.51	
Therapy, n (%)			0.278
Chemotherapy	152 (89.94)	27.16	
Palliative care	17 (10.06)	23.21	
Lymphocyte count (10^9^/L), n (%)			0.539
≤7.22	103 (60.95)	26.4	
> 7.22	36 (39.05)	27.13	
Basophil count (10^9^/L), n (%)			
≤ 0.01	126 (74.56)	26.3	0.333
> 0.01	33 (25.44)	27.79	
NLR, n (%)			< 0.001
≤ 5	130 (76.92)	26.48	
> 5	39 (23.08)	28.39	
KPS score, n (%)			< 0.001
60	11 (6.5)	19.01	
70	45 (26.63)	21.17	
80	93 (55.01)	29.4	
90	20 (11.83)	32.95	
Histology, n (%)			0.715
Adenocarcinoma	73 (43.2)	26.77	
Squamous cell carcinoma	77 (45.56)	28.19	
Small cell carcinoma	17 (10.06)	22.53	
Other	2 (1.18)	24	
Disease stage			0.019
I–II	12 (7.1)	36.58	
III–IV	157 (92.9)	26.22

**Table 3:** Comparison of peripheral leukocyte levels in lung cancer patients and normal controls

Subgroups (10^9^/L)	Normal group	Lung cancer group	P value
White blood cell count	6.471 ± 0.149	7.031 ± 0.141	0.001
Neutrophil count	3.628 ± 0.118	4.849 ± 0.122	< 0.001
Lymphocyte count	2.210 ± 0.069	1.415 ± 0.041	< 0.001
Mononuclear cell count	0.619 ± 0.114	0.599 ± 0.020	0.879
Eosinophils	0.161 ± 0.012	0.156 ± 0.011	0.491
Basophils	0.045 ± 0.008	0.011 ± 0.001	0.027
Neutrophils/lymphocytes	1.774 ± 0.082	4.067 ± 0.178	< 0.001

Most patients were aged > 55 years (74.56%, 126/169), were female (24.26%, 41/169), and were smokers (26.63%, 45/169). Based on the standard for tumour, node, metastasis (TNM) stage, 12 patients (7.1%) were in stage I + II, and 157 patients (92.9%) were in stage III + IV. There were 73 cases of lung adenocarcinoma, 77 cases of lung squamous cell carcinoma, 17 cases of small cell carcinoma, and two other diseases among the lung cancer samples. Patients were dichotomized according to a prespecified cut-off value of an NLR ≥ 5 vs. < 5, as an NLR ≥ 5 has been previously validated as being associated with overall survival (OS) in patients with lung cancer [33]. In addition, the cut-off value of OS was set to the median value of 27.

### Determination of causal relationships between the NLR and OS

To test causal relationships between variables, patients were subdivided into two groups (N = 130–39). The higher NLR group had an NLR > 5 (n = 39, 23.08%), and the lower NLR group had an NLR ≤ 5 (n = 130, 76.92%). We took patient *u* at random in the lower NLR group and selected *v* with similar conditions, which means similar age, same sex, same cancer type and so on, with *u* from the control set for pairing. Then, the *outcome (u, v)* variables and overall evaluation parameter δ of each matched pair were calculated to determine whether there was a causal relationship between the research variables. All patients were divided into 35 matching pairs, including 22 positive pairs, 10 negative pairs, and 3 zero pairs. Therefore, the value of δ was 34.286%, which provided strong evidence of the causality between the NLR and OS. In addition, we used the sign test (95% confidence interval) to ensure the credibility and reliability of the results. The mean and variance were 34.5 and 4.153 throughout, respectively. The model’s Z statistic (2.04656) was larger than the *Z* $\alpha /2$ (1.96) statistic, implying a causal relationship between the two.

### Evaluation of the causal effect

**Fig. 3:**
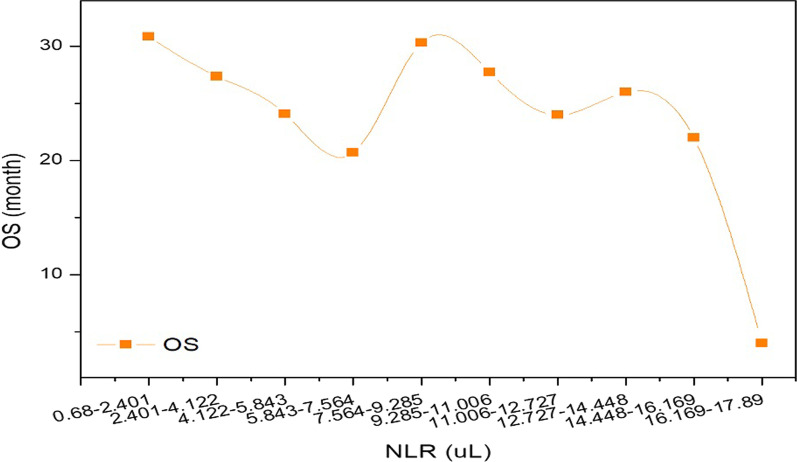
Relationship between baseline NLR and OS

Based on the previous results, the NLR was regularly altered with the change in OS, while our data analysis (Fig. 3) was contradictory. Therefore, according to the method in the above exposition, we analysed the observed data from different perspectives.

From Table 3, we can draw some conclusions. The peripheral white blood cell count, neutrophil count, and NLR of lung cancer patients were significantly higher than those of healthy controls (P < 0.05), while the lymphocyte count and basophil count were lower than those of healthy controls (P < 0.05), and the difference was statistically significant. In the high NLR group and the low NLR group, we counted the number of patients with each clinicopathological datum in each group and compared them with the X^2^ test. The results showed that the difference in the NLR in the KPS score of lung cancer patients before treatment was statistically significant (P < 0.05); there was no significant difference in clinical data, such as classification (P > 0.05). In addition, we also performed univariate analysis on peripheral blood leukocytes and clinicopathological data of lung cancer patients. The results showed that smoking, tumour stage, KPS score, and NLR were all factors affecting the survival of lung cancer patients. The age, sex, cancer type, white blood cell count, neutrophil count, lymphocyte count, and basophil count of lung cancer patients were not associated with the survival and prognosis of the patients.

**Fig. 4:**
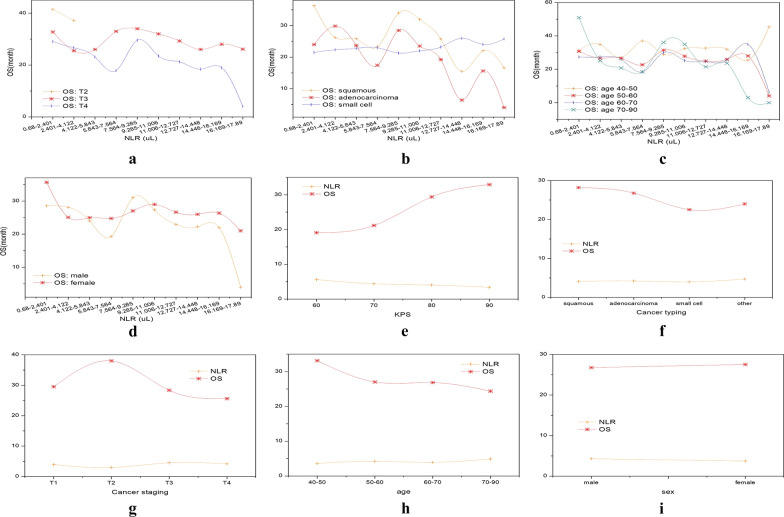
Relationship between baseline NLR and OS under different type, cancer stages (**a**, **g**), cancer type (**b**, **f**), age (**c**, **h**), sex (**d**, **i**), and KPS score (**e**). Among them, **a**–**d** is a confounding factor analysis, and **e**–**i** is an intermediate value analysis. Due to the lack of data, we used SPSS to fill in the missing data, but for the categories with fewer data (the second stage of cancer), we adopted the omission and merge method

**Fig. 5:**
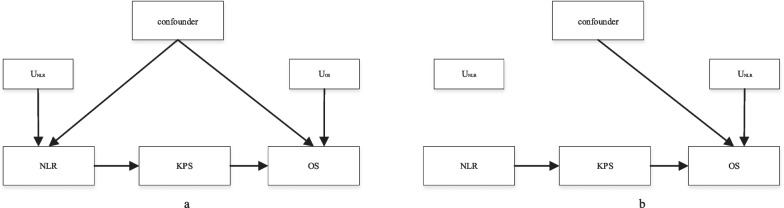
Causal graph between the NLR and survival time. A graphical model representing the causal effects of the NLR on OS; confounders are an unknown element, and KPS score is a mediator(**a**). An intervention on the model in Fig. 4a that changes the NLR in the population (**b**)

Combining Tables 1, 2, 3 and Fig. 4, we find that OS decreases significantly as the NLR increases in Fig. 4e. We can explain this phenomenon through theoretical common sense. The NLR is an inflammatory marker with high sensitivity and specificity, and it represents the balance between inflammatory activator neutrophils and inflammatory regulator lymphocytes. The essence of an elevated NLR is the increase in neutrophils and the decrease in lymphocytes. The higher the NLR is, the more pronounced the imbalanced state and the more serious the inflammation. Severe inflammation may lead to a decline in the patient's mobility, deterioration of their disease, and limit the patients' self-care ability, which will lead to a decrease in KPS score. Our conclusion was also confirmed through the literature [20, 34–36]. The causal graph between the NLR and OS is shown in Fig. 5a. The modified graphical model (denoted in alphabetical letters as G$\overline{x}$), which is necessary for us to quantify the causal relationship between them, representing an intervention in the model in Fig. 5a is shown in Fig. 5b.

In this study, *X* = 1 stands for the lower NLR (defined by the previous), *Z* stands for the KPS scores of patients, and *Y* = 1 stands for the higher OS (defined by the median OS). To evaluate the effect of interventions in the study, we need to eliminate the do operation in $P (Y |do (X = x))$ and estimate the difference $P(Y = 1|do(X = 1)) - P(Y = 1|do(X = 0))$. The derivation process is as follows:

$$P\left(Y=y|do\left(X=x\right)\right)$$

2 $$=\sum_{Z}P(Y|{\text{do}}(X),Z)P(Z|do(X))$$

3 $$=\sum_{Z}P\left(Y|do\left(X\right),do(Z)\right)P\left(Z|do(X)\right)$$

4 $$=\sum_{Z}P\left(Y|do\left(X\right),do\left(Z\right)\right)P\left(Z|X\right)$$

5 $$=\sum_{Z}P\left(Y|do(Z)\right)P\left(Z|X\right)$$

6 $$=\sum_{X{^{\prime}}}\sum_{Z}P\left(Y|do\left(Z\right),X{^{\prime}}\right)P\left(X{^{\prime}}|do(Z)\right)P\left(Z|X\right)$$

7 $$=\sum_{X{^{\prime}}}\sum_{Z}P\left(Y|Z,X{^{\prime}}\right)P\left(X{^{\prime}}|do(Z)\right)P\left(Z|X\right)$$

8 $$=\sum_{z}P(Z=z,X)\sum_{X}P\left(Y|X=x,Z=z\right)P(X=x)$$

9 $$P\left(Y=y|do\left(X=x\right)\right) =\sum_{z}P(Z=z,X)\sum_{X}P\left(Y|X=x,Z=z\right)P(X=x)$$

Formulas (2) and (6) were constructed using the Bayesian formula; Formulas (3), (4) and (7) were constructed using Role 2; and Formulas (5) and (8) were constructed using Role 3. Bringing the experimental data into Eq. (9) to obtains:

10 $$P\left(Y=1|do\left(X=0\right)\right)=0.173077*0.047337+0.453963*0.195266+0.699023*0.426036+0.864253*0.100592=0.481582$$

11 $$P(Y=1|do(X=1))=0.173077*0.017751+0.453963*0.071006+0.699023*0.12426+0.864253*0.017751=0.137509$$

Thus, comparing the effect of NLR-higher (*X* = 1) to the effect of NLR-lower (*X* = 0), we obtain:

12 $$ACE=P\left(Y=1|do\left(X=0\right)\right)-P(Y=1|do(X=1))\text{=0.3}{44073}$$

giving a clear positive advantage to NLR-lower. The causal association between the NLR and OS is 34.4%; that is, under the same survival environment, patients with lower NLRs have a higher survival rate.

### Accuracy of the result

In medicine, a cohort study is often undertaken to obtain evidence to refute the existence of a suspected association between cause and effect, and failure to refute a hypothesis often strengthens confidence in it. Crucially, the cohort is identified before the appearance of the disease under investigation, which aids greatly in studying causal associations [37, 38]. In survival analysis, the hazard ratio (HR) [39] is the ratio of the hazard rates corresponding to the condition described by the two levels of the explanatory variable. In addition to capturing information about the entire Kaplan–Meier (KM) survival curve, the HR also provides an estimate of the relative efficacy between treatment groups (e.g., HR = 0.75 for the OS endpoint, which means that the mortality rate of the experimental group is reduced by approximately 25% compared to the control group). Therefore, we selected nine papers on cohort studies on the NLR and the prognosis of lung cancer patients. The relative ranges of the NLR and OS causality were determined by HRs (0.2291, 0.6487). Our result agrees with the literature findings and with real-world data, which further proves that our method is correct.

## Discussion

There have been many methods for investigating potential causal relationships between disease and treatment options in recent years, which can be categorized into two main types: experimental studies and observational studies. Researchers control the experimental conditions and evaluate the intervention effects in experimental studies. Due to the nature of experimental studies, financial resources, human resources, and patients' ethical considerations, the researchers cannot fully control the exposure of the research participants. Therefore, many of the findings are from observational studies, specifically from case–control studies. Regardless of the method adopted, the results in most cases only determine causal relationships. They cannot intervene with observational data, let alone answer the questions needed for medical research.

This work presents a new experimental strategy called quasi-intervention for evaluating the effects of specific treatments without clinical trials by using a causal inference method. The quasi-intervention consisted of a QED [5–7], sign test [40] and hypothetical intervention [8–11]. We used the QED to establish the causal association between the intervening and outcome variables and used a sign test to ensure the reliability of the results. Hypothetical intervention can quantify the causal effect without simulating the intervention, which saves money and is easy to implement to evaluate the accuracy of the quasi-intervention by quantifying the causal effect between the NLR and OS. Our results showed that a low or decreased NLR leads to a significant improvement in OS. This result was consistent with a previous study, proving that our method is correct.

Compared with other observational studies, our study is unique in the following aspects:

1. The method incorporates as many confounding factors as possible into the study, making the experiment more rigorous. A QED considers known confounders in the data, and hypothetical interventions consider potential confounders.
2. The method relaxes the conditions of the research environment, uses a series of ingenious, intelligent observation methods to simulate the actual experiment, and combines the cause-and-effect diagram to obtain the actual intervention effect.
3. This method can complete some intervention experiments that cannot actually be completed for factors such as patient's obesity, blood pressure, and smoking status. It allows us to determine causal effects in nonexperimental studies.

There are some limitations to this study. First, our data was retrospectively collected and selected from the hospital, so there might be selection bias or recall bias. Second, a causal graph critically influences the obtained results, and it is affected by assumptions and confounding factors. Although we excluded some confounders, unmeasured confounders still impacted the results. These factors would introduce more bias and limit the method's generalizability to a broader patient population.

## Conclusion

In summary, this work provides a new method for evaluating the effect of interventions that can be applied in the fields of clinical medicine. The presented results from our method could provide a causal effect between disease and treatment options. We believe that the proposed method can be applied to clinically relevant research to obtain more results.

## Data Availability

The datasets generated and/or analysed during the current study are not publicly available due to the sensitive nature of the questions asked in this study, but are available from the corresponding author on reasonable request.

## Abbreviations

ACE: Average causal effect
QED: Quasi-experimental design
NLR: Neutrophil-to-lymphocyte ratio
OS: Overall survival
KPS: Karnofsky performance status

## References

[1] Pearl J, Mackenzie D (2018). The book of why: the new science of cause and effect. Science.

[2] Rubin DB (1974). B: Estimating causal effects of treatments in randomized and nonrandomized studies. J Educ Psychol.

[3] Dimasi JA, Grabowski HG, Hansen RW (2016). Innovation in the pharmaceutical industry: new estimates of R&D costs. J Health Econ.

[4] Brady H, Collier D, Sekhon JS. The Neyman–Rubin model of causal inference and estimation via matching methods. 2008.

[5] Harris AD, Bradham DD, Baumgarten M, Zuckerman IH, Perencevich EN (2004). The use and interpretation of quasi-experimental studies in infectious diseases. Clin Infect Dis.

[6] Marinescu IE, Lawlor PN, Kording KP (2018). Quasi-experimental causality in neuroscience and behavioural research. Nat Hum Behav.

[7] Harris AD, Lautenbach E, Perencevich E (2005). A systematic review of quasi-experimental study designs in the fields of infection control and antibiotic resistance. Clin Infect Dis.

[8] Pearl J (2016). Lord's paradox revisited—(Oh Lord! Kumbaya!). J Causal Inference.

[9] Robins MJ (2008). Causal models for estimating the effects of weight gain on mortality. Int J Obes.

[10] Pearl J (1998). Graphs, causality, and structural equation models. Sociol Methods Res.

[11] Pearl J (2014). Interpretation and identification of causal mediation. Psychol Methods.

[12] Jin F, Han AQ, Shi F, Kong L, Yu JM (2016). The postoperative neutrophil-to-lymphocyte ratio and changes in this ratio predict survival after the complete resection of stage I non-small cell lung cancer. Oncotargets Ther.

[13] Xie XH, Liu JJ, Yang HT, Chen HJ, Zhou SJ, Lin H, Liao ZY, Ding Y, Ling LT, Wang XW (2019). Prognostic value of baseline neutrophil-to-lymphocyte ratio in outcome of immune checkpoint inhibitors. Cancer Investig.

[14] Forget P, Machiels JP, Coulie PG, Berliere M, Poncelet AJ, Tombal B, Stainier A, Legrand C, Canon JL, Kremer Y (2013). Neutrophil: lymphocyte ratio and intraoperative use of ketorolac or diclofenac are prognostic factors in different cohorts of patients undergoing breast, lung, and kidney cancer surgery. Ann Surg Oncol.

[15] Abravan A, Salem A, Price G, Faivre-Finn C, van Herk M (2013). Effect of systemic inflammation biomarkers on overall survival after lung cancer radiotherapy: a single-center large-cohort study. Acta Oncol.

[16] Lan H, Zhou L, Chi D, Zhou Q, Tang X, Zhu D, Yue J, Liu B (2017). Preoperative platelet to lymphocyte and neutrophil to lymphocyte ratios are independent prognostic factors for patients undergoing lung cancer radical surgery: a single institutional cohort study. Oncotarget.

[17] Liu D, Jin J, Zhang L, Li L, Song J, Li W (2018). The neutrophil to lymphocyte ratio may predict benefit from chemotherapy in lung cancer. Cell Physiol Biochem.

[18] Seong YW, Han SJ, Jung W, Jeon JH, Cho S, Jheon S, Kim K (2019). Perioperative change in neutrophil-to-lymphocyte ratio (NLR) is a prognostic factor in patients with completely resected primary pulmonary sarcomatoid carcinoma. J Thorac Dis.

[19] Cedrés S, Torrejon D, Martínez A, Martinez P, Navarro A, Zamora E, Mulet-Margalef N, Felip E (2012). Neutrophil to lymphocyte ratio (NLR) as an indicator of poor prognosis in stage IV non-small cell lung cancer. Clin Transl Oncol.

[20] Diem S, Schmid S, Krapf M, Flatz L, Born D, Jochum W, Templeton AJ, Früh M (2017). Neutrophil-to-Lymphocyte ratio (NLR) and Platelet-to-Lymphocyte ratio (PLR) as prognostic markers in patients with non-small cell lung cancer (NSCLC) treated with nivolumab. Lung Cancer.

[21] Morgan MS, Hendry DF. The foundations of econometric analysis: the foundations of econometric analysis. 1995.

[22] Carozzi FM, Bisanzi S, Carrozzi L, Falaschi F, Lopes-Pegna A, Mascalchi M, Picozzi G, Peluso M, Sani C, Greco L (2017). Multimodal lung cancer screening using the ITALUNG biomarker panel and low dose computed tomography. Results of the ITALUNG biomarker study. Int J Cancer.

[23] Chunshan S, Haiyang Y, Dejun S, Lili M, Zhaohui T (2015). Cisplatin-loaded polymeric nanoparticles: characterization and potential exploitation for the treatment of non-small cell lung carcinoma. Acta Biomater.

[24] Su Y, Hu Y, Wang Y, Xu X, Yuan Y, Li Y, Wang Z, Chen K, Zhang F, Ding X (2017). A precision-guided MWNT mediated reawakening the sunk synergy in RAS for anti-angiogenesis lung cancer therapy. Biomaterials.

[25] Freddie B, Jacques F, Isabelle S, Rebecca SL (2018). Global cancer statistics 2018: GLOBOCAN estimates of incidence and mortality worldwide for 36 cancers in 185 countries. CA Cancer J Clin.

[26] Zhang Z, Zeng K, Zhao S, Zhao Y, Hou X, Luo F, Lu F, Zhang Y, Zhou T, Ma Y (2019). Pemetrexed/carboplatin plus gefitinib as a first-line treatment for EGFR-mutant advanced nonsmall cell lung cancer: a Bayesian network meta-analysis. Ther Adv Med Oncol.

[27] Siegel RL, Miller KD, Jemal A (2018). Cancer statistics, 2018. CA Cancer J Clin.

[28] Sung H, Ferlay J, Siegel RL, Laversanne M, Soerjomataram I, Jemal A, Bray F (2021). Global cancer statistics 2020: GLOBOCAN estimates of incidence and mortality worldwide for 36 cancers in 185 countries. CA Cancer J Clin.

[29] Diem S, Schmid S, Krapf M, Flatz L, Born D, Jochum W, Templeton AJ, Fruh M (2017). Neutrophil-to-Lymphocyte ratio (NLR) and Platelet-to-Lymphocyte ratio (PLR) as prognostic markers in patients with non-small cell lung cancer (NSCLC) treated with nivolumab. Lung Cancer.

[30] Bagley SJ, Kothari S, Aggarwal C, Bauml JM, Alley EW, Evans TL, Kosteva JA, Ciunci CA, Gabriel PE, Thompson JC (2017). Pretreatment neutrophil-to-lymphocyte ratio as a marker of outcomes in nivolumab-treated patients with advanced non-small-cell lung cancer. Lung Cancer.

[31] He JR, Shen GP, Ren ZF, Qin H, Cui C, Zhang Y, Zeng YX, Jia WH (2012). Pretreatment levels of peripheral neutrophils and lymphocytes as independent prognostic factors in patients with nasopharyngeal carcinoma. Head Neck.

[32] Templeton AJ, McNamara MG, Šeruga B, Vera-Badillo FE, Aneja P, Ocaña A, Leibowitz-Amit R, Sonpavde G, Knox JJ, Tran B (2014). Prognostic role of neutrophil-to-lymphocyte ratio in solid tumors: a systematic review and meta-analysis. J Natl Cancer Inst.

[33] Sarraf KM, Belcher E, Raevsky E, Nicholson AG, Goldstraw P, Lim E (2009). Neutrophil/lymphocyte ratio and its association with survival after complete resection in non–small cell lung cancer. J Thorac Cardiovasc Surg.

[34] Mandaliya H, Jones M, Oldmeadow C, Nordman II (2019). Prognostic biomarkers in stage IV non-small cell lung cancer (NSCLC): neutrophil to lymphocyte ratio (NLR), lymphocyte to monocyte ratio (LMR), platelet to lymphocyte ratio (PLR) and advanced lung cancer inflammation index (ALI). Transl Lung Cancer Res.

[35] Russo A, Russano M, Franchina T, Migliorino MR, Aprile G, Mansueto G, Berruti A, Falcone A, Aieta M, Gelibter A (2020). Neutrophil-to-lymphocyte ratio (NLR), platelet-to-lymphocyte ratio (PLR), and outcomes with nivolumab in pretreated non-small cell lung cancer (NSCLC): a large retrospective multicenter study. Adv Ther.

[36] Liu J, Li S, Zhang S, Liu Y, Ma L, Zhu J, Xin Y, Wang Y, Yang C, Cheng Y (2019). Systemic immune-inflammation index, neutrophil-to-lymphocyte ratio, platelet-to-lymphocyte ratio can predict clinical outcomes in patients with metastatic non-small-cell lung cancer treated with nivolumab. J Clin Lab Anal.

[37] Power C (2006). Elliott, Jane: Cohort profile: 1958 British birth cohort (National Child Development Study). Int J Epidemiol.

[38] Schlesselman J (1974). Sample size requirements in cohort and case-control studies of disease. Am J Epidemiol.

[39] Spruance SL, Reid JE, Grace M, Samore M (2004). Hazard ratio in clinical trials. Antimicrob Agents Chemother.

[40] Diebold FX, Mariano RS (1995). Comparing predictive accuracy. J Bus Econ Stat.

